# Supplementary material for: Choice of vector and surgical approach enables efficient cochlear gene transfer in nonhuman primate
Source: Nat Commun. 2022 Mar 15;13:1359. doi: 10.1038/s41467-022-28969-3 (PMC8924271; doi:10.1038/s41467-022-28969-3)
Supplement: Supplementary file 1 — Supplementary information [file 41467_2022_28969_MOESM1_ESM.pdf]

Supplementary Materials:

Supplementary Table 1. Clinical history of rhesus macaques treated with viral vectors.

Animal ID (AAV serotype)	Previous history and treatment	Post-operative complications	Post-operative treatment
RA3009 (AAV1)	None	None	None
RA3109 (AAV1)	None	None	None
RA3120 (Anc80)	Fistula in left cheek. Treated with antibiotics, NSAIDs*, anxiolytic drugs	None	None
RA3131 (Anc80)	None	Facial paresis with incomplete left eye closure	Partial tarsorrhaphy
RA3128(Anc80)	None	None	None

*NSAIDs = nonsteroidal anti-inflammatory drugs.

Supplementary Table 2. Serum biochemistry values from non-human primates injected with AAV1 and Anc80. The values outside the reference range were highlighted in red (above) and blue (below).

	RA3009 (Day 0)	RA3009 (Day 7)	RA3109 (Day 0)	RA3109 (Day 7)	RA3120 (Day 0)	RA3120 (Day 7)	RA3131 (Day 0)	RA3131 (Day 7)	RA3128 (Day 0)	RA3128 (Day 14)
ALP (0-704 U/L)	482	355	---	464	250	289	732	613	388	387
AST (1-46 U/L)	23	22	---	26	23	29	23	28	31	32
ALT (1-59 U/L)	25	40	---	37	14	41	25	61	26	33
CK (0-1596 U/L)	219	282	---	649	173	60	135	284	414	149
Albumin (3.2-4.8 g/dL)	4.9	3.4	---	4.7	3.2	3.6	5.6	5.0	5.5	5.1
Total bilirubin (0-0.4 mg/dL)	0.3	0.2	---	0.1	0.0	0.2	0.4	0.2	0.3	0.2
Total protein (5.9-8 g/dL)	7.1	5.2	---	7.5	6.0	6.5	8.7	7.8	8.3	7.7
Globulin (2.1-3.7 g/dL)	2.2	1.8	---	2.8	2.8	2.9	3.1	2.8	2.8	2.6
Bilirubin-conjugated (0.00-0.25 mg/dL)	0.1	0.0	---	0.0	0.0	0.0	0.0	0.0	0.0	0.0
BUN (9-23 mg/dL)	15	14	---	19	19	19	20	18	21	17
Creatinine (0.7-1.4 mg/dL)	0.6	0.3	---	0.7	0.6	0.5	0.6	0.6	0.7	0.6
Cholesterol (69-205 mg/dL)	133	96	---	126	96	105	188	149	185	195
Glucose (33-95 mg/dL)	69	55	---	83	74	84	75	105	97	118
Calcium (7.6-10.7 mg/dL)	10.6	7.8	---	9.5	8.2	8.3	11.7	11.1	11.5	10.7
Phosphorus (0.9-8 mg/dL)	7.1	4.8	---	6.8	6.3	4.6	6.6	6.7	8.3	6.8
Bicarbonate TCO2 (5-35 mmol/L)	23	20	---	22	24	24	25	25	24	25
Chloride (91-121 mmol/L)	131	94	---	108	110	100	130	126	137	128
Potassium (3.1-5.1 mmol/L)	4.5	3.0	---	4.3	4.2	3.1	4.1	3.9	4.5	4.0
ALB/GLOB ratio (0.866-1.942)	2.2	1.9	---	1.7	1.1	1.2	1.8	1.8	2.0	2.0
Sodium (140-152 mmol/L)	173	128	---	153	148	139	178	176	184	174
Bilirubin-unconjugated (0.00-0.2 mg/dL)	0.2	0.2	---	0.1	0.0	0.2	0.4	0.2	0.3	0.2
Na/K ratio (27-40)	38	43	---	36	35	45	43	45	41	44

Supplementary Table 3. Vector genome copy numbers in selected tissues. eGFP genome copy numbers/diploid cell DNA. Genomic DNA was isolated from the indicated tissues. The data are shown as mean values ± SD. ND = not detectable. Source data are provided as a Source Data file.

GC/cell	RA3009	RA3109	RA3120	RA3131	RA3128
Cerebellum	0.00011±0.00009	0.00075±0.00035	ND	0.00025±0.00022	0.00011±0.00009
Spinal cord	0.00020±0.00033	0.01059±0.00042	ND	0.00046±0.00003	0.00013±0.00011
Frontal lobe	0.00012±0.00011	0.00929±0.00260	0.00034±0.00002	0.00181±0.00132	ND
Temporal lobe	0.00022±0.00023	0.00224±0.00055	0.00006±0.00010	0.00122±0.00017	ND
Parietal lobe	0.00022±0.00010	0.00750±0.00259	ND	0.00073±0.00044	0.00013±0.00011
Occipital lobe	0.00016±0.00027	0.00727±0.00051	ND	0.00064±0.00034	ND

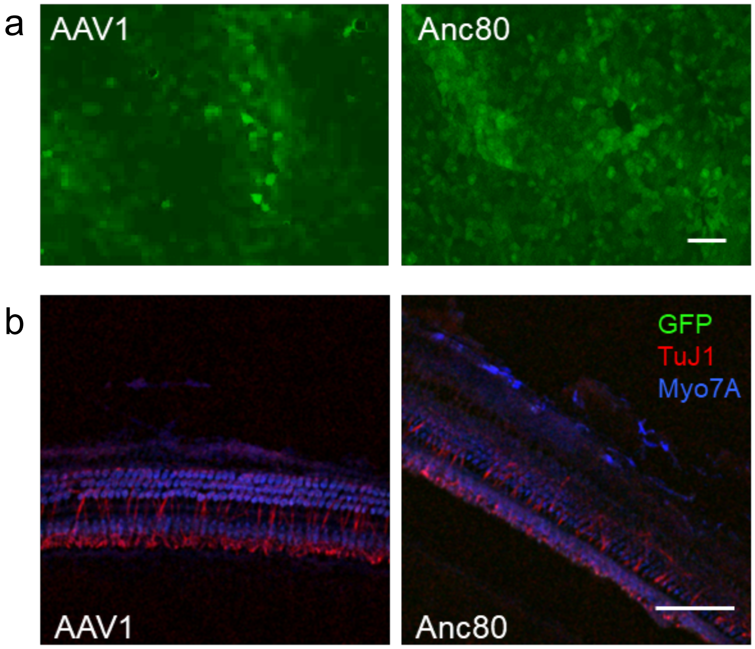

Supplementary Figure 1. (**a**) Representative images of eGFP (green) fluorescence signal detected across the liver after intravenous injection of the same dose of Anc80L65.CB7.eGFP (n=2) and AAV1.CB7.eGFP (n=2) in young adult C57BL/6 male wild-type mice. (**b**) Representative whole mount images of cochlear middle turns of the contralateral uninjected ears of NHP after unilateral intracochlear administration through the round window membrane (n=2 animals for AAV1, and n=3 animals for Anc80L65). No eGFP signal was detected in the contralateral ears after administering Anc80 or AAV1. Whole mounts of cochlear middle turns were co-stained with Myo7A (blue, for hair cells) and TuJ1 (red, for neuronal structures). Scale bar, 100 µm.

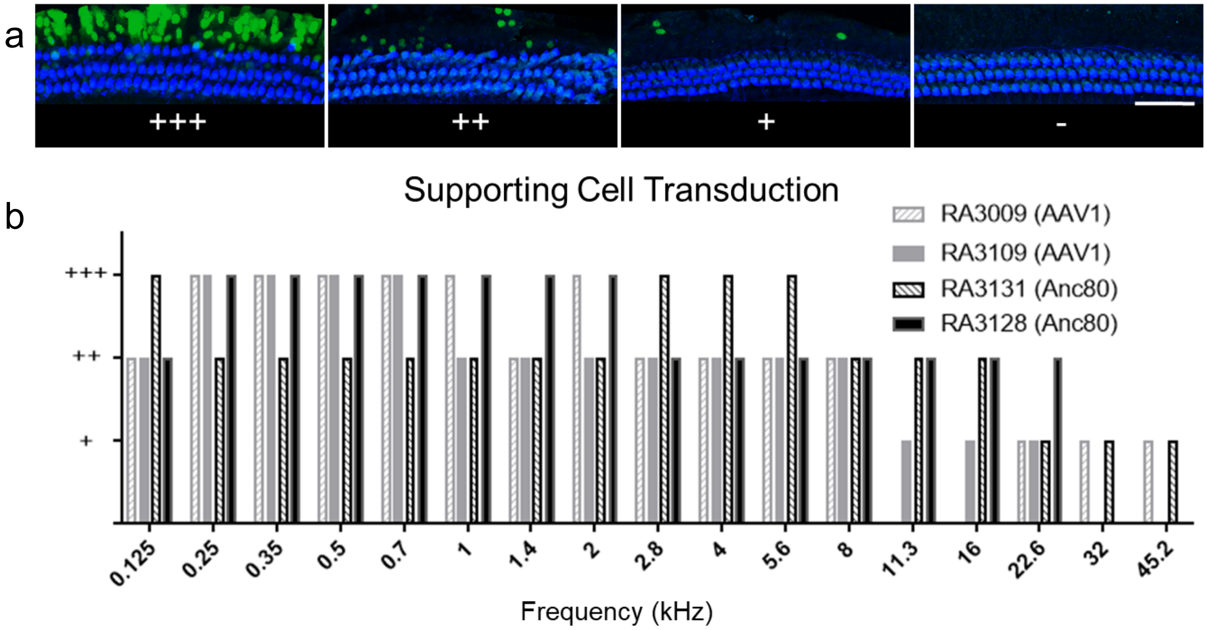

Supplementary Figure 2. *In vivo* supporting cell transduction after ipsilateral intracochlear administration through the round window membrane in rhesus macaques. (**a**) Representative images of cochlear whole mounts from animals injected with AAV1 (n=2) and Anc80L65 (n=2). The areas of the spiral limbus and supporting cells were assessed qualitatively using a scale from "3 plus signs" (strongest signal) to "minus sign" (no expression). Green, eGFP-positive supporting cells. Blue, Myo7A-stained (outer) hair cells. Scale bar, 50 µm. (**b**) Qualitative assessment of eGFP-positive supporting cells per frequency region along the length of the cochlea, with a gradual increase in expression from the base to the apex. Identical confocal microscope settings were used to obtain all images. No obvious differences were detected between the AAV serotypes. Source data are provided as a Source Data file.

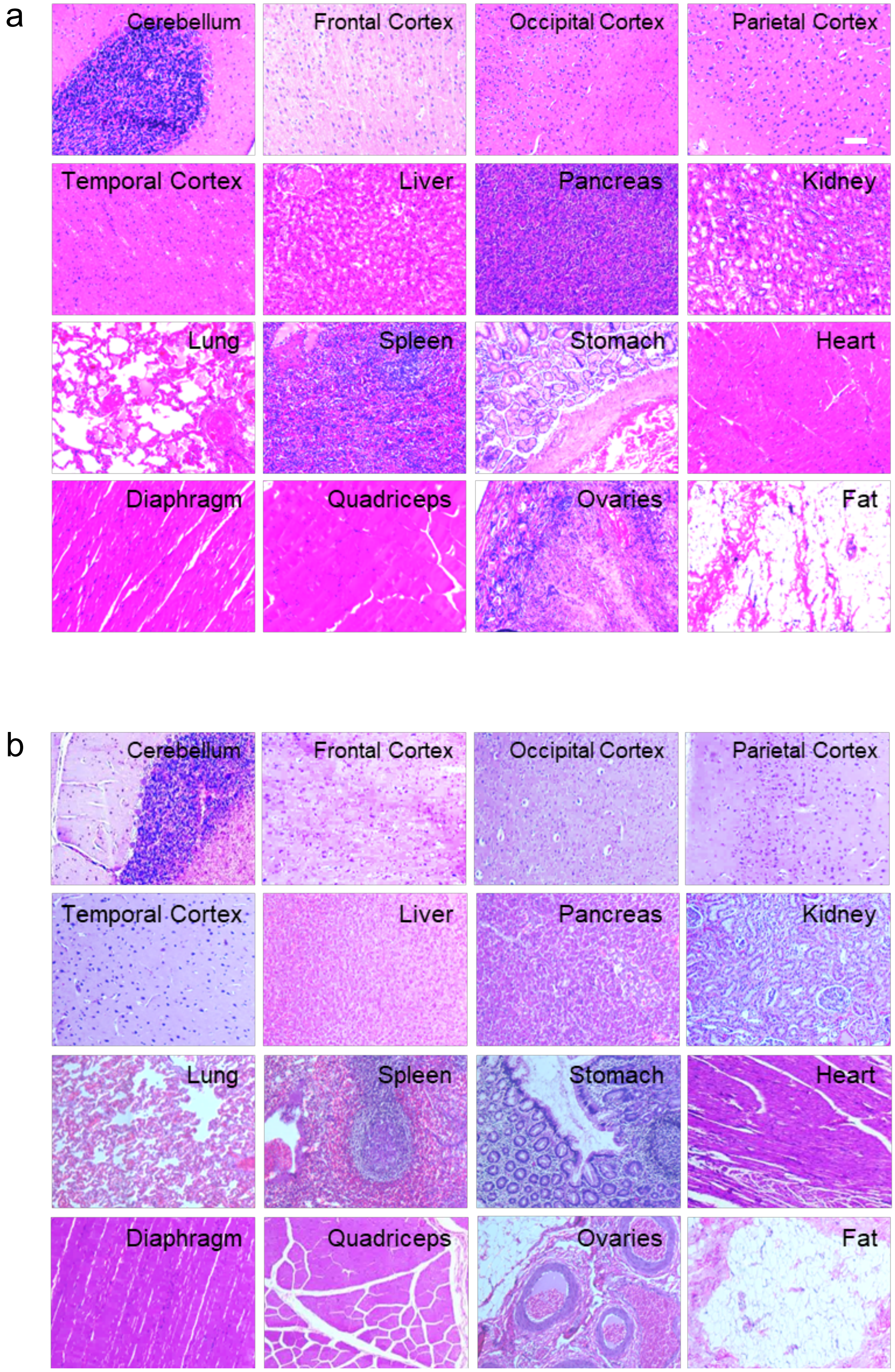

Supplementary Figure 3. Representative images of different CNS regions and organs from two rhesus macaques injected through the round window membrane with Anc80 (**a**) and AAV1 (**b**). No signs of toxicity or inflammation were found. No differences were found between both animals. Tissues were stained with Hematoxylin & Eosin following standard procedures. Scale bar, 100 µm.

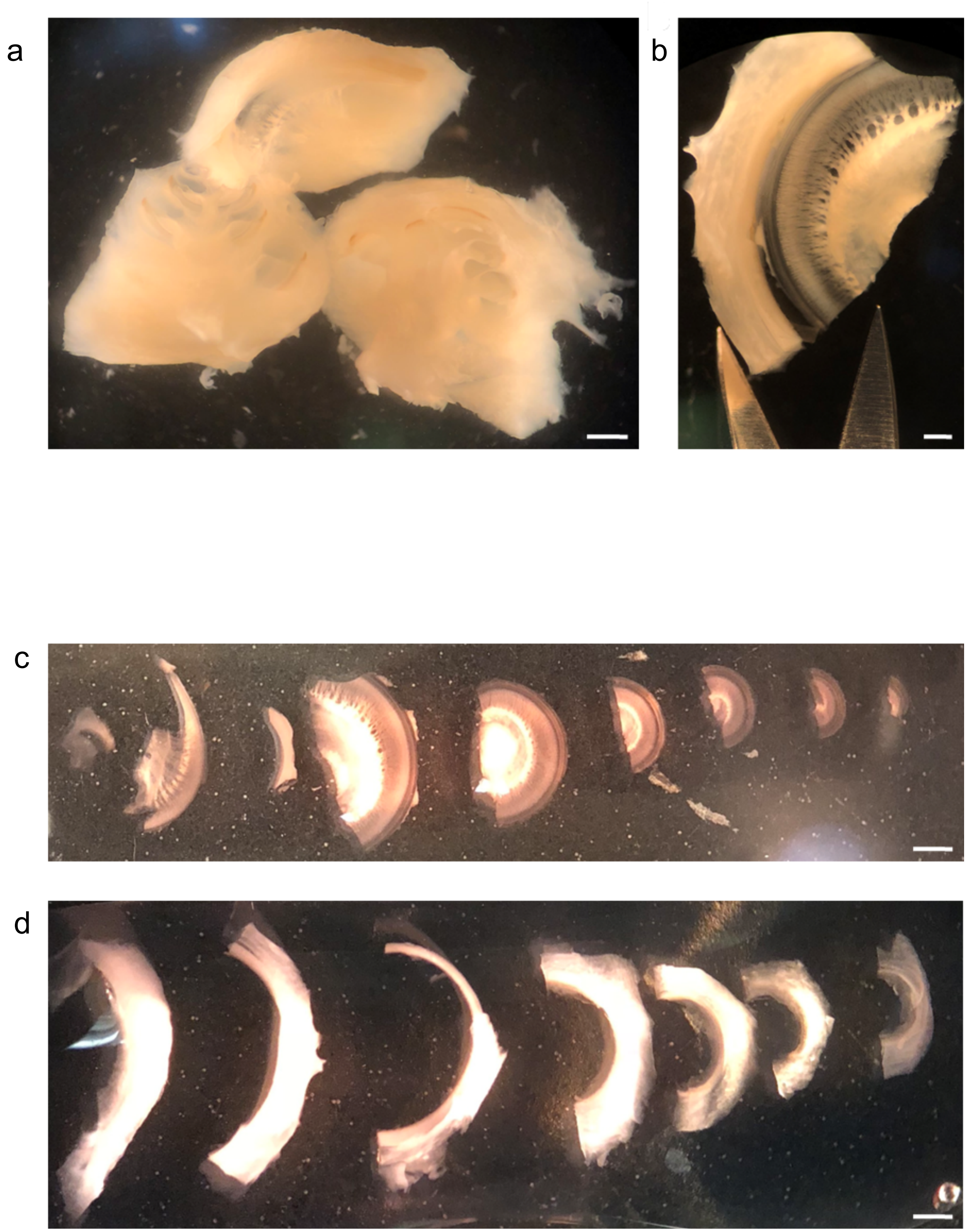

Supplementary Figure 4. Dissection steps to process monkey inner ears. (**a**) After the initial dissection, (**b**) microscissors were used to separate the lateral wall and organ of Corti area for every individual cochlear turn. (**c**) Representative images (n=5 monkeys) of extracted organ of Corti and (**d**) lateral wall sections for one cochlea from basal (left) to apical (right). Scale bar, 1 mm in a, c, d and 500 µm for b.

Supplementary Movie 1. Representative video of the surgical procedure on a left ear in a rhesus macaque. See also Fig 2. A mastoidectomy was performed to identify the posterior wall of the external auditory canal anteriorly and the tegmen mastoideum superiorly. An extended facial recess approach was used to expose the round window membrane after skeletonizing the facial nerve. A fenestration in the oval window was performed, and the AAV vector was microinjected through the round window membrane.
