# Supplementary material for: Choice of vector and surgical approach enables efficient cochlear gene transfer in nonhuman primate
Source: Nat Commun. 2022 Mar 15;13:1359. doi: 10.1038/s41467-022-28969-3 (PMC8924271; doi:10.1038/s41467-022-28969-3)
Supplement: Supplementary file 2 — Reporting Summary [file 41467_2022_28969_MOESM2_ESM.pdf]

nature portfolio

Corresponding author(s): Luk H. Vandenberghe
Last updated by author(s): Jul 29, 2021

Reporting Summary

Nature Portfolio wishes to improve the reproducibility of the work that we publish. This form provides structure for consistency and transparency in reporting. For further information on Nature Portfolio policies, see our Editorial Policies and the Editorial Policy Checklist.

Statistics

For all statistical analyses, confirm that the following items are present in the figure legend, table legend, main text, or Methods section.

n/a	Confirmed	
☐	☒	The exact sample size (n) for each experimental group/condition, given as a discrete number and unit of measurement
☐	☒	A statement on whether measurements were taken from distinct samples or whether the same sample was measured repeatedly
☒	☐	The statistical test(s) used AND whether they are one- or two-sided<br>*Only common tests should be described solely by name; describe more complex techniques in the Methods section.*
☒	☐	A description of all covariates tested
☒	☐	A description of any assumptions or corrections, such as tests of normality and adjustment for multiple comparisons
☐	☒	A full description of the statistical parameters including central tendency (e.g. means) or other basic estimates (e.g. regression coefficient) AND variation (e.g. standard deviation) or associated estimates of uncertainty (e.g. confidence intervals)
☒	☐	For null hypothesis testing, the test statistic (e.g. F, t, r) with confidence intervals, effect sizes, degrees of freedom and P value noted<br>*Give P values as exact values whenever suitable.*
☒	☐	For Bayesian analysis, information on the choice of priors and Markov chain Monte Carlo settings
☒	☐	For hierarchical and complex designs, identification of the appropriate level for tests and full reporting of outcomes
☒	☐	Estimates of effect sizes (e.g. Cohen's d, Pearson's r), indicating how they were calculated

Our web collection on statistics for biologists contains articles on many of the points above.

Software and code

Policy information about availability of computer code

Data collection	Zeiss Axio Imager M2 Zen Imaging Software Version 2.3, QuantaSoft Software Version 1.7, Leica TCS SP5 Confocal Software (LAS AF Version 3.2), Gen5™ Microplate Reader and Imager Software Version 2.0
Data analysis	Microsoft Office 365 Excel Version 2110, GraphPad Prism Version 9.1.2, Image J Version 1.53

For manuscripts utilizing custom algorithms or software that are central to the research but not yet described in published literature, software must be made available to editors and reviewers. We strongly encourage code deposition in a community repository (e.g. GitHub). See the Nature Portfolio guidelines for submitting code & software for further information.

Data

Policy information about availability of data

All manuscripts must include a data availability statement. This statement should provide the following information, where applicable:
- Accession codes, unique identifiers, or web links for publicly available datasets
- A description of any restrictions on data availability
- For clinical datasets or third party data, please ensure that the statement adheres to our policy

Source data analyzed are provided with this paper, cell (IHC and SC) quantifications and biodistribution data generated in this study are provided in the Source Data. Raw confocal/microscopy images and datasets are available from the corresponding author on reasonable request.

March 2021

Field-specific reporting

Please select the one below that is the best fit for your research. If you are not sure, read the appropriate sections before making your selection.

- [x] Life sciences
- [ ] Behavioural & social sciences
- [ ] Ecological, evolutionary & environmental sciences

For a reference copy of the document with all sections, see nature.com/documents/nr-reporting-summary-flat.pdf

Life sciences study design

All studies must disclose on these points even when the disclosure is negative.

Sample size	Given the exploratory and qualitative nature of the study, and to reduce the number of non-human primates used during the study,no sample size calculation was performed.
Data exclusions	No data exclusion were performed.
Replication	All the viral vectors were titared at least three times to verify the vector genome concentration.<br>Immunological assays and tissue biodistribution experiments were run in triplicates of each tissue or fluid per animal. The average of the triplicates is presented in the paper. Results are presented as individual results from each animal<br>NHP experiments were performed in two animals for AAV1 and 3 animals for Anc80. Given the exploratory and descriptive nature of the study, results are presented individually per animal. Quantification of e-GFP positive cells(IHC and SC) in NHP was successfully replicated by two independent researchers.<br>Mouse intravenous injections and liver GFP signal analysis was performed in 2 animals per group; and mice intracochlear injections and cochlear analysis were performed in 3 animals per group. Similar results were found in animals from the same group, although, as expected for in vivo experiments, variability was observed. Results are presented as representative individual animals, but no group analyses were performed.
Randomization	Animals were enrolled in the study based on a clean health record, no experimental treatment history, and serum neutralizing antibody titers against AAV1 or Anc80L65 below 1:10. They were assigned by simple randomization to the study groups based on their neutralizing antibody titers (low or no detectable) to the AAV serotype selected.
Blinding	Quantification of eGFP-positive cells of every mapped frequency region was manually quantified along the length of the cochlea by two independent researchers blinded to the treatment and group allocation during the data collection and analysis.

Reporting for specific materials, systems and methods

We require information from authors about some types of materials, experimental systems and methods used in many studies. Here, indicate whether each material, system or method listed is relevant to your study. If you are not sure if a list item applies to your research, read the appropriate section before selecting a response.

Materials & experimental systems

n/a	Involved in the study	
☐	☒	Antibodies
☐	☒	Eukaryotic cell lines
☒	☐	Palaeontology and archaeology
☐	☒	Animals and other organisms
☒	☐	Human research participants
☒	☐	Clinical data
☒	☐	Dual use research of concern

Methods

n/a	Involved in the study	
☒	☐	ChIP-seq
☒	☐	Flow cytometry
☒	☐	MRI-based neuroimaging

Antibodies

Antibodies used	Neurofilament H (#AB5539, Merck Millipore); Myosin 7A (Myo7A, #25-6790 Proteus Biosciences); GFP [9F9.F9] (#ab1218, Abcam); Iba-1 (#019-19741, Wako Chemicals).<br>Secondary antibodies: Goat anti-Mouse IgG1 Alexa Fluor 488 (#A-21121, Thermo Fisher Scientific), Goat anti-Chicken IgY (H+L) Alexa Fluor 568 (#A-11041, Thermo Fisher Scientific), Donkey anti-Rabbit IgG (H+L) Alexa Fluor 647 (#A-31573, Thermo Fisher Scientific), Goat anti-Chicken IgY (H+L) Alexa Fluor 647 (#A-21449, Thermo Fisher Scientific), and Goat anti-Rabbit IgG (H+L) Alexa Fluor 647 (#A-21245, Thermo Fisher Scientific)
Validation	Neurofilament H (#AB5539)- Validated for Bovine, Human, Mouse,Pig,Rat,Feline. Applications: ICC, IHC, IH(P), WB (https://www.emdmillipore.com/US/en/product/Anti-Neurofilament-H-Antibody,MM_NF-AB5539#overview)<br>Myosin 7A (Myo7A, #25-6790)- Validated for Human, Mouse, Rat, Pig, Avian and Amphibian. Applications: IF, WB (http://

www.proteus-biosciences.com/product/view/myosin-viia-248.aspx)
GFP [9F9.F9] (#ab1218, Abcam)- Species independent. Applications: WB, IHC-Fr, Sandwich ELISA, ICC/IF (https://www.abcam.com/gfp-antibody-9f9f9-ab1218.html)
Iba-1 (#019-19741)- Validated for Human, Mouse and Rat. Applications: ICC, IHC(Frozen) (https://labchem-wako.fujifilm.com/us/product/detail/W01W0101-1974.html)
Goat anti-Mouse IgG1 Alexa Fluor 488 (#A-21121, Thermo Fisher Scientific) Applications: WB, IHC, IHC-P and F, ICC, Flow, IP (https://www.thermofisher.com/antibody/product/Goat-anti-Mouse-IgG1-Cross-Adsorbed-Secondary-Antibody-Polyclonal/A-21121)
Goat anti-Chicken IgY (H+L) Alexa Fluor 568 (#A-11041, Thermo Fisher Scientific) Applications: WB, IHC, IHC-P and F, ICC, Flow (https://www.thermofisher.com/antibody/product/Goat-anti-Chicken-IgY-H-L-Secondary-Antibody-Polyclonal/A-11041)
Donkey anti-Rabbit IgG (H+L) Alexa Fluor 647 (#A-31573, Thermo Fisher Scientific) Applications: WB, IHC, IHC-P and F, ICC, Flow (https://www.thermofisher.com/antibody/product/Donkey-anti-Rabbit-IgG-H-L-Highly-Cross-Adsorbed-Secondary-Antibody-Polyclonal/A-31573)
Goat anti-Chicken IgY (H+L) Alexa Fluor 647 (#A-21449, Thermo Fisher Scientific) Applications: WB, IHC, IHC-P and Free-floating, ICC (https://www.thermofisher.com/antibody/product/Goat-anti-Chicken-IgY-H-L-Secondary-Antibody-Polyclonal/A-21449)
Goat anti-Rabbit IgG (H+L) Alexa Fluor 647 (#A-21245, Thermo Fisher Scientific) Applications: WB, IHC, IHC-P -F and Free-floating, ICC, Flow, DB (https://www.thermofisher.com/antibody/product/Goat-anti-Rabbit-IgG-H-L-Highly-Cross-Adsorbed-Secondary-Antibody-Polyclonal/A-21245)

Eukaryotic cell lines

Policy information about cell lines

Cell line source(s)	HEK293 cells
Authentication	None, commercial cells
Mycoplasma contamination	Cells were tested for mycoplasma contamination and they were negative.
Commonly misidentified lines (See ICLAC register)	No commonly misidentified cell lines were used in this study

Animals and other organisms

Policy information about studies involving animals; ARRIVE guidelines recommended for reporting animal research

Laboratory animals	Mouse- Mus musculus, strain C57BL/6 male (6-8 weeks), and strain CD1 female and male (4 day old), housing conditions are stated in the methods.<br>Rhesus Macaques- Macaca mulatta, female (3 to 4 years old), housing conditions are stated in the methods.
Wild animals	No wild animals were used in this study
Field-collected samples	No field collected samples were used in this study
Ethics oversight	All experiments were approved by the Institutional Animal Care and Use Committee at Schepens Eye Research Institute as well as the Institutional Biosafety Committee.

Note that full information on the approval of the study protocol must also be provided in the manuscript.
