# Supplementary material for: Choice of vector and surgical approach enables efficient cochlear gene transfer in nonhuman primate
Source: Nat Commun. 2022 Mar 15;13:1359. doi: 10.1038/s41467-022-28969-3 (PMC8924271; doi:10.1038/s41467-022-28969-3)
Supplement: Supplementary file 4 — Description of Additional Supplementary Files [file 41467_2022_28969_MOESM4_ESM.pdf]

Title: Supplementary Movie 1.
Description: Representative video of the surgical procedure on a left ear in a rhesus macaque. See also Fig 2. A mastoidectomy was performed to identify the posterior wall of the external auditory canal anteriorly and the tegmen mastoideum superiorly. An extended facial recess approach was used to expose the round window membrane after skeletonizing the facial nerve. A fenestration in the oval window was performed, and the AAV vector was microinjected through the round window membrane.
